# Relationships between Behavioural Addictions and Psychiatric Disorders: What Is Known and What Is Yet to Be Learned?

**DOI:** 10.3389/fpsyt.2017.00053

**Published:** 2017-04-07

**Authors:** Vladan Starcevic, Yasser Khazaal

**Affiliations:** ^1^Discipline of Psychiatry, Sydney Medical School Nepean, University of Sydney, Sydney, NSW, Australia; ^2^Department of Psychiatry, University of Geneva, Geneva, Switzerland; ^3^Research Center, Montreal University Institute of Mental Health, Montreal, QC, Canada

**Keywords:** behavioural addiction, pathological gambling, problematic Internet use, Internet addiction, problematic online gaming, compulsive sexual behaviour disorder, compulsive buying, exercise addiction

## Abstract

This article provides a narrative review of the relationships between several behavioural addictions [pathological gambling, problematic Internet use (PIU), problematic online gaming, compulsive sexual behaviour disorder, compulsive buying, and exercise addiction] and psychiatric disorders. Associations between most behavioural addictions and depressive and anxiety disorders are strong and seem relatively non-specific. Strong links with substance use disorders may support the notion that some people are more prone to addictive behaviours, regardless of whether these involve substances or problematic activities. Other associations seem relatively specific, for example, those between PIU/online gaming and attention-deficit/hyperactivity disorder, between compulsive buying on the one hand and eating disorders and hoarding on the other hand and between exercise addiction and eating disorders. The quality of the research varies, but most studies suffer from methodological limitations, including a cross-sectional or correlational design, non-representative study populations, small sample sizes, reliance on self-report assessment instruments, diverse diagnostic criteria, and conceptual heterogeneity of most behavioural addictions. Due to these limitations, generalisability of the findings is questionable and the direction of causality, if any, is unknown in the relationships between behavioural addictions and psychiatric disorders. Regardless of the aetiological uncertainty, these relationships often call for a modified treatment approach. Prospective studies are needed to clarify the longitudinal relationships between behavioural addictions and psychiatric disorders.

## Introduction

Behavioural addictions are a controversial concept (1, 2), sitting between the realms of impulse control disorders and substance use disorders. One of the issues surrounding behavioural addictions has been the extent to which they can be conceptualised as distinct and independent entities. Related to this has been a question of the relationships between various behavioural addictions and other psychopathology.

This article provides a narrative review of the relationships between a number of behavioural addictions and psychiatric disorders. Considering that many repetitive and problematic behaviours have been deemed to constitute behavioural addictions (3), the article addresses only those that have frequently or relatively consistently been endorsed as such (1): pathological gambling, problematic Internet use (PIU), problematic online gaming, compulsive sexual behaviour disorder (CSBD), compulsive buying, and exercise addiction.

## Pathological Gambling (Gambling Disorder)

The key feature of pathological gambling, renamed gambling disorder in DSM-5 (4), is the loss of control over urges to gamble, with numerous adverse consequences in multiple domains of functioning. The status of pathological gambling as a behavioural addiction is least disputed, but its classification as an impulse control disorder has been proposed for ICD-11 due to its confirmed or presumed close links not only with substance use disorders, but also with impulse control disorders, major depressive disorder (MDD), and mania (5).

Indeed, numerous studies have demonstrated that pathological gambling is often related to various forms of psychopathology. One systematic review and meta-analysis of population studies (6) reported that nicotine dependence was the most common co-occurring condition (60.1%) in “problem and pathological gamblers,” followed by any substance use disorder (alcohol abuse/dependence and/or drug abuse/dependence and/or nicotine dependence; 57.5%), any mood disorder (MDD, dysthymia and bipolar disorder/manic episodes; 37.9%), any anxiety disorder (panic disorder with or without agoraphobia, generalised anxiety disorder, social anxiety disorder and specific phobia; 37.4%), and antisocial personality disorder (28.8%).

Similar rates of co-occurring psychiatric disorders were repor-ted in treatment-seeking samples of individuals with problem/pathological gambling. A systematic review and meta-analysis of co-occurring psychiatric disorders in treatment-seeking problem gamblers found that rates were 74.8% for any current “Axis I” disorder and 75.5% for any lifetime “Axis I” disorder (7). In the same study, the most frequent current co-occurring disorder was nicotine dependence (56.4%), followed by MDD (29.9%), any mood disorder (23.1%), any alcohol or substance use disorder (22.2%), any anxiety disorder (17.6%), and attention-deficit/hyperactivity disorder (ADHD; 9.3%). The most frequent lifetime co-occurring disorders were MDD (54.3%) and any alcohol or substance use disorder (47.0%).

Aetiological implications of these findings remain unclear because of the methodological limitations of most studies, especially their cross-sectional design. Even a review of the longitudinal studies has not been able to shed more light on the issue of the extent to which the specific psychiatric disorders predispose or lead to pathological gambling as opposed to being its consequence (8). This review reported that impulsivity was an important, underlying factor in many cases of pathological gambling, facilitating both the onset of “problem” gambling and progression from less severe to more severe forms of pathological gambling, often in conjunction with depression and substance use disorders.

Treatment implications are similarly unclear, although there is some indication that co-occurring conditions do not necessarily interfere with the outcome of standard treatments for pathological gambling (9). Still, the presence of co-occurring psychiatric disorders may indicate poorer outcome (10) and usually calls for a modified treatment approach to pathological gambling so that these disorders are also targetted. However, there are no guidelines as to how to do this in clinical practice. If the patient is severely depressed or suicidal, the priorities seem clear, but in cases of mild to moderate depression co-occurring with pathological gambling, it is uncertain whether the two conditions should best be treated concomitantly or sequentially. Similar dilemmas exist when substance use disorders co-occur with pathological gambling; in such cases, one option may be to address the underlying propensity to addictive behaviours instead of focussing on these behaviours separately (6).

## PIU (Internet Addiction)

Problematic Internet use or “Internet addiction” is a controversial entity, not listed in DSM-5. It is increasingly regarded as inadequate because addiction to a medium or delivery mechanism (such as the Internet) is considered conceptually untenable and because PIU is too heterogeneous, encompassing a variety of online behaviours such as gaming, gambling, social networking, shopping, and sexual activities (11).

The rates of psychiatric disorders and symptoms in individuals with PIU vary from one study to another. The most common co-occurring psychiatric disorders and symptoms are depression (in up to 77.8% of individuals with PIU) (12), substance abuse (up to 55%) (13), anxiety disorders (up to 50%) (14), social anxiety disorder (up to 45%) (13), symptoms of sleep disturbance (up to 38.6%) (15), and ADHD (up to 32.2%) (16). In one review (17), one systematic review (18) and one meta-analysis (19), the strongest associations with PIU were reported for depression/depressive disorders (17–19) and ADHD/symptoms of ADHD (17–19), followed by substance use disorder (17), alcohol abuse (19), and anxiety/anxiety symptoms (18, 19). Strong associations between PIU and social anxiety disorder (17) and between PIU and obsessive–compulsive symptoms (18) were reported inconsistently. Significant predictors of PIU included ADHD (17) and hyperactivity/inattention (20), depression/depressive disorder (17, 20), social anxiety disorder (17), anxiety (20), hostility (17), conduct problems (20), and suicidal ideation and suicide attempts (20). Patterns of co-occurring disorders in PIU may be different in adults and adolescents, with a tendency for personality disturbance, ADHD, conduct disorder, dissociative symptoms/disorders, social anxiety disorder, obsessive–compulsive disorder (OCD), and insomnia to be reported more frequently in adolescents (21).

The interpretation of these findings is difficult because of the aforementioned conceptual problems with PIU, making it possible that various problematic Internet-related behaviours are associated distinctly with different psychopathological entities, as suggested by some preliminary studies (22, 23). Almost all the studies conducted to date have been cross-sectional or correlational, leaving open the question of causality, if any, in the relationships between PIU and various disorders. Most findings were based on self-report instruments, symptom checklists and/or questionnaires with single items, and various diagnostic criteria for PIU/Internet addiction were used across the studies. Finally, studies were conducted in different populations (e.g., adolescents, high school or university students, outpatients, only males) from different countries and using different sample sizes and various recruitment strategies. All these raise a question about the generalisability of the findings.

Research limitations notwithstanding, practical implications pertain to the treatment of individuals with PIU. Despite limited evidence, it seems reasonable to use pharmacotherapy and/or psychotherapeutic approaches for co-occurring disorders, regardless of whether they may be a cause or a consequence of PIU or related to PIU in some other way. Thus, administering stimulants in the presence of ADHD may be warranted, and one study of children with both ADHD and “Internet video game addiction” reported a significant improvement with methylphenidate on the Internet Addiction Scale, a measure of addictive Internet use (24). Likewise, a study of individuals with MDD and “excessive online game play” showed that bupropion significantly reduced both the scores on the Internet Addiction Scale and levels of depression (25).

## Problematic Online Gaming [Online Gaming Addiction, Internet Gaming Disorder (IGD)]

Problematic online gaming (or online gaming addiction) was initially viewed as a manifestation of PIU/Internet addiction and studies of PIU/Internet addiction often included individuals with this specific pattern of problematic online behaviour. In DSM-5, IGD was introduced as a condition for further study, reflecting that problematic online gaming is not synonymous with PIU (26) and that there is insufficient evidence to grant IGD the status of an official diagnosis.

Data on co-occurring psychiatric disorders in individuals with problematic online gaming/IGD are limited both because there has been a tendency to study individuals with this behavioural pattern as “Internet addicts” and because the diagnostic criteria for IGD appeared only in 2013, with the publication of DSM-5. Due to the heterogeneity of the samples with PIU/Internet addiction, extrapolating from the findings of studies of PIU/Internet addiction to apply to problematic online gaming/IGD should be avoided.

Problematic online gaming has been associated with increased levels of depression (27–31), anxiety (27), and social anxiety (28, 29) and with symptoms of inattention and ADHD (32). The only study thus far that investigated a co-occurring diagnosis of ADHD reported that 39.1% of young adults with IGD met the diagnostic criteria for this condition and that impulsivity and hostility mediated the association between IGD and ADHD (33).

Methodological limitations noted in the section on PIU/Internet addiction also apply to the few studies of psychopatho-logy associated with problematic online gaming/IGD. In addition, findings may differ depending on the type of game played. The uncertain direction of causality, if any, in the relationships between problematic online gaming/IGD and psychiatric disorders makes it difficult to be more specific about their treatment implications beyond the usual suggestion that the presence of co-occurring conditions may call for a modified treatment approach.

## CSBD (Hypersexual Disorder, Sex Addiction)

Compulsive sexual behaviour disorder (CSBD) is the term proposed for ICD-11 for a condition also known as hypersexual disorder and sex addiction. It has not been included in DSM-5. As with most other behavioural addictions, it is uncertain whether CSBD is a true addiction or an impulse control disorder. The hallmark of the condition is a poorly controlled sex drive, so that short-term sexual gratification overrides potential long-term harm. CSBD is heterogeneous with regards to sexual behaviours, sexual orientation, online or offline sexual activities, and consequences ranging from relationship breakdown to sexual offending.

Two studies conducted in small clinical samples of mostly males with CSBD reported lifetime rates of any “Axis I” disorder of 83% (34) and 100% (35). They reported the following co-occurrence rates for the specific disorders: any anxiety disorder up to 96% (35), any mood disorder up to 71% (35), any substance use disorder up to 71% (35), any sexual dysfunction up to 46% (35), any personality disorder up to 46% (35), any impulse control disorder up to 38% (35), and OCD up to 14% (34). Rates of antisocial and borderline personality disorders were low in these studies. One meta-analytic review of the correlational studies found a moderate positive correlation (*r* = 0.34) of CSBD and depressive symptoms (36).

There are important clinical implications of these findings, despite the methodological problems with the aforementioned studies, their questionable representativeness, and uncertainty about the nature of the relationships between CSBD and psychopathology. Considering the high frequency with which various psychiatric disorders co-occur with CSBD, it is imperative to properly assess all individuals with CSBD for the presence of these disorders and address them in treatment. Relatively strong associations with certain forms of psychopathology may be counterintuitive (e.g., between CSBD on the one hand and depression, social anxiety, and sexual dysfunction on the other hand), while weak associations with other disorders may be surprising (e.g., between CSBD and more severe personality disorders and bipolar disorder). This underscores a need to shift an exclusive diagnostic and therapeutic focus away from the manifestations of CSBD.

## Compulsive Buying (Shopping Addiction)

Compulsive buying refers to irresistible and overwhelming urges to buy objects that are usually not needed. Insight into the senselessness of excessive shopping and its detrimental consequences is insufficient to prevent further shopping. Com-pulsive buying is not an official diagnosis in the DSM or ICD system, and its optimal conceptualisation remains uncertain, although it is usually considered a behavioural addiction.

Similar to other behavioural addictions, compulsive buying tends to be frequently associated with various psychiatric disorders. Five studies examined the patterns of co-occurrence with psychiatric disorders, but except for one (37), others were conducted in small samples, ranging from 20 to 44 subjects (38–41). The largest study reported a lifetime rate of any co-occurring “Axis I” disorder of 89.5% in individuals with compulsive buying (37). Any mood disorder was the most common lifetime diagnosis (in up to 95%) (39), followed by any anxiety disorder (up to 80%) (39), MDD (up to 62.6%) (37), any personality disorder (59%) (40), any impulse control disorder (up to 40%) (39), any eating disorder (up to 35%) (39), any substance use disorder (up to 30%) (40), OCD (up to 18.7%) (37), posttraumatic stress disorder (13.5%) (37), social anxiety disorder (9.1%) (41), and bipolar disorder (4.7%) (37). The relationships between compulsive buying on the one hand and some impulse control disorders, behavioural addictions, and eating disorders on the other hand are “bidirectional” so that individuals with pathological gambling (42), exercise addiction (43), and binge eating disorder, bulimia nervosa, or morbid obesity (44, 45) seem to be more likely to have higher rates of compulsive buying. A recent study of Internet shoppers found an association between compulsive buying, trait anxiety, and OCD symptoms (46).

Compulsive buying appears to have a relatively specific relationship with hoarding. In one study, 62% of individuals with compulsive buying in a clinical sample also exhibited pathological hoarding (47), whereas compulsive buying was significantly more common in OCD individuals with hoarding than in those without hoarding (48). In DSM-5, this relationship was recognised by introducing a specifier “with excessive acquisition” for the newly created diagnostic category of hoarding disorder.

Although the cross-sectional design of most studies precludes a conclusion about the nature of the relationships between compulsive buying and various psychiatric disorders, the frequency with which they co-occur has implications for assessment and treatment of compulsive buying. Focussing only on compulsive buying would run the risk of overlooking co-occurring disorders in need of therapeutic attention; treatment of these conditions might have a beneficial impact on the outcome of treatment of compulsive buying.

## Exercise Addiction (Exercise Dependence)

Similar to most other behavioural addictions, exercise addiction has been a controversial concept. This is reflected in its original designation as a “positive addiction” (49) because no harm was envisaged as a consequence of excessive exercising. After exercise addiction crossed the boundary of a disorder (although not from the DSM or ICD perspective), a distinction was made between its primary and secondary forms. The former is construed as a condition in which the exercise itself is the main or sole objective, whereas weight loss is the objective of the latter, with exercising being only the means of achieving that objective (50). However, this distinction is not always easy because individuals with primary exercise addiction are often preoccupied with their weight, dieting, and body image and the primary motivation for exercising may not be clear.

Not surprisingly, exercise addiction appears to have a strong relationship with eating disorders, especially bulimia nervosa (43), with secondary exercise addiction being in fact a manifestation of an eating disorder. Also, features of exercise addiction and eating disorders overlap with those of muscle dysmorphia. Exercise addiction has been considered an obsessive–compulsive spectrum disorder (50); it has also been linked with symptoms of anxiety and depression, whereby repetitive or compulsive exercise may be driven by a need to alleviate anxiety and depression (51) or avoid distress or unpleasant emotional states (52). However, these relationships have not been sufficiently investigated and little is known about their implications.

## Discussion

While there is plenty of evidence that behavioural addictions frequently co-occur with various psychiatric disorders, the quality of that evidence is such that the central questions remain unanswered: When do certain psychiatric disorders predispose to or cause certain behavioural addictions and when do these disorders occur as a consequence of behavioural addictions? Are they related in some other ways, e.g., via a common underlying factor? Only prospective studies will shed more light on this important issue and ultimately clarify whether or not and under which circumstances some behavioural addictions may be conceptualised as distinct and independent psychopathological entities.

Table 1 shows that associations between behavioural addictions and certain psychiatric disorders are strong and relatively non-specific (i.e., they are not confined only to some behavioural addictions). This applies particularly to depressive and anxiety disorders and may suggest that behavioural addictions are a maladaptive way of coping with the primary states of depression or anxiety or that depressive and anxiety disorders occur as a consequence of various problems associated with behavioural addictions.

**Table 1:** **Associations between behavioural addictions and psychiatric disorders and symptoms**.

	Any mood disorder	Depressive disorders	Bipolar disorder	Any anxiety disorder	Social anxiety disorder	OCD	Hoarding	PTSD	Any substance use disorder	Nicotine dependence	Any impulse control disorder	ADHD	Any personality disorder	ASPD	Any eating disorder	Sexual dysfunction	Sleep disorder
Pathological gambling	+++	++++	+	+++	++	+		++	++++	++++	+^a^	+		+++			
Problematic Internet use		+++	++^b^	+++	++	++		+^b^	+++		+^b^	+++			+^b^		++^b^
Problematic online gaming		+++^b^		+^b^	++^b^							+++^b^					
Compulsive sexual behaviour disorder	+++	+++	+	++++	++	+		++^b^	++++		++		+++	+	+	+++^b^	
Compulsive buying	++++	++++	+	++++	+	+	++^b^	++^b^	++		++		++++^b^		++		
Exercise addiction		?		?		?									++^b^		

*^a^This association pertains to kleptomania and intermittent explosive disorder*.

*^b^These estimates of association are based on an insufficient number of studies*.

*++++ = very strong association (≥50% rate in at least 1 meta-analysis/systematic review or in at least 2 studies); +++ = strong association (≥25% rate in at least 1 meta-analysis/systematic review or in at least 2 studies); ++ = moderate association (≥10% rate in at least 1 meta-analysis/systematic review or in at least 2 studies); + = relatively weak association (<10% rate in at least 1 meta-analysis/systematic review or in at least 1 study); ? = uncertain association*.

*OCD, obsessive–compulsive disorder; PTSD, posttraumatic stress disorder; ADHD, attention-deficit/hyperactivity disorder; ASPD, antisocial personality disorder*.

A common association with substance use disorders was also suggested by a study showing that individuals with alcohol use disorder had a significantly higher rate of various behavioural addictions compared to those without alcohol use disorder (53). This may support the notion about the propensity towards addictive behaviours, regardless of whether these involve psychoactive substances or certain repetitive and problematic activities. However, this does not necessarily mean that behavioural addictions should be conceptualised as addictions because poor impulse control may be a hallmark of these disorders.

Some associations seem relatively specific, such as those between pathological gambling and nicotine dependence, between PIU/problematic online gaming and ADHD and sleep disorder, between CSBD and sexual dysfunction, between compulsive buying on the one hand and bulimia-like eating disorders and hoarding on the other hand, and between exercise addiction and eating disorders (Table 1). Caution is needed here because the apparently specific nature of some relationships may reflect a failure to study conditions such as nicotine dependence and ADHD across all behavioural addictions. Nevertheless, further study of these relationships may clarify the ways in which some forms of psychopathology (especially ADHD) interact with certain behavioural addictions.

Behavioural addictions seem to have a weaker association with OCD than with other disorders, suggesting that they should not be conceptualised as obsessive–compulsive spectrum disorders. Certain behavioural addictions have a relationship with bipolar disorder, but it is important to ascertain whether manifestations of pathological gambling, CSBD, and compulsive buying can be better explained by bipolar disorder (i.e., behaviour during manic episodes) because that would preclude the diagnoses of these behavioural addictions.

The present review is limited by its narrative nature, i.e., the fact that it is not a systematic review. However, to the best of our knowledge, this is the first attempt to examine relationships with psychiatric disorders across various behavioural addictions. A need for further research and for a systematic review in this area cannot be overemphasised. Only studies that are better designed and devoid of the aforementioned methodological problems will allow expansion of our knowledge about the relationships between behavioural addictions and other psychopathology and better understanding of their treatment implications.

## Author Contributions

Both the authors contributed equally to the design, writing, and final approval of this article.

## Conflict of Interest Statement

The authors have no commercial or financial relationships that could be construed as a potential conflict of interest.
